# News and Notes

**Published:** 1905-07

**Authors:** 


### *NEWS AND NOTES.*

THE London Medical Society has awarded its Fothergillian prize for 1905 to Sir F. Treves.

DR. F. W. MCRAE is in attendance on the Portland meeting of the American Medical Association.

DR. A. L. FOWLER has been appointed physician to the Federal prison vice Dr. J. C. Swann, resigned.

DR. DAVID LINN EDSALL has been elected assistant professor of medicine in the University of Pennsylvania.

DR. A. H. BUCKMASTER and Dr. W. C. Christian have resigned from the faculty of the University of Virginia.

THE commencement exercises of the Tabernacle Infirmary Training School for Nurses will take place July 3.

THE first doctor's degree conferred on a woman by the University of Marburg has been won by a Japanese lady, Miss Tada Urata, of Kumamoto.

A NUMBER of ladies belonging to the highest society in Rome have formed a committee to devise means for the relief of sufferers from ringworm, which is very prevalent in the Province of Rome.

THE International Association of Climatologists will meet in annual session at Portland, Oregon, July 11 to 14, 1905, under the presidency of Dr. John Davis Hartley, of Sacramento. The Secretary is Mr. Chas. H. Peck, 259 William street, New York.

THE Public Health Reports state that the government has ordered the closing of the large schools, and has put in force many measures to prevent the introduction of plague in Santiago. The intensity of the epidemic in Pisaqua has somewhat diminished, though a dispatch dated March 14, states that the population is greatly reduced.

---

SIR JAMES PAGET has followed up the lives of 1,000 medical students who had joined the medical school at St. Bartholomew's Hospital, with the following result: Twenty-three met with distinguished success, 66 met with considerable success, 507 met with fair success, 124 met with very limited success, 56 failed, 96 discontinued medical studies while in pupilage, 41 died during pupilage, and 87 died within twelve years of commencing practice.

---

THE widwives of Italy are shortly to meet in a National Congress to be held at Milan. Intimations of adhesions have been received from the Midwives' Societies of Turin, Genoa, Milan, Bologna, Florence, Rome, and Cremona. The municipality of the city and the Council of the Province of Naples have promised their support. Addresses will be delivered by Professors A. Guzzoni, of Messina; E. Pestalozza, of Florence; and L. Bossi, of Genoa.

---

IT is reported from Paris by an exchange that Dr. Albert Robin, who a short time ago described good results in the treatment of pneumonia by the intravenous injection of metallic silver and manganese in the collodial state, to which preparations he has given the name of metallic ferments, has been treating meningitis by the same method in the Hospital Beaujon. The injections are said to be followed by a decrease in the temperature and improvement in the general condition.

MENINGITIS has been raging several months in epidemic form in Silesia. Much alarm exists among the people, especially the laboring population, and many have fled from the district. The disease is beginning to make its appearance in other parts of the country. The epidemic in Silesia has assumed such an alarming form that the Prussian Government has been petitioned regarding the measures taken to check it. About 1,200 cases have occurred in the Oppeln District, with about 50 per cent. of fatalities.

---

THE surgeon-general of the army has sent out letters to chief surgeons calling their attention to the disregard of kitchen economy at army hospitals. It is found that too liberal advantage is taken of the existence of the special diet fund, and that many patients who might very properly subsist on the regular ration, are permitted to draw upon the special diet. It provides that hereafter there shall be precaution against extravagances of this kind. A circular has been issued by the chief surgeon of the Department of Dakota, based on the admonition of the surgeon-general.

---

A CONGRESS of doctors, says *American Medicine*, from all parts of Russia was held in Moscow recently, ostensibly to consider means for combatting an expected epidemic of cholera, but its proceedings assumed considerable of the character of a political assemblage. Resolutions were adopted declaring that under existing political and economic conditions it would be practically impossible to fight an epidemic of disease, as doctors and inspectors would not be safe from attack by ignorant peasants angered by the prevalent distress. The resolutions proceeded to demand changes in the system of allotment of lands, reform in taxation, the convoking of an elective constituent assembly, and other articles of the advanced liberal programme. Since that time a number of those taking part in the proceedings have been arrested.

THE medical statistics of the French army for the year 1902, lately published, are based, says an exchange, upon an effective of 485,207 men. The total number received in infirmaries and military hospitals gave an average of 594 men per 1,000, this large number being slightly less than in the previous year. The figures given refer to France. In Algeria and Tunis the proportion rose to 634 per 1,000, but this was notably less than in the previous year. The returns of those received in hospitals and infirmaries are based upon somewhat different conditions, officers being included in one case and not in the other, but the figures are nevertheless sufficiently close to give a good notion of the terrible rate of morbidity. In the case of officers the proportion is very much less. The deaths were 4.24 per 1,000 in France and 8.3 in Algeria and Tunis, these figures being much lower than in previous records.

---

IT is reported that plans are being formed by the various fraternal associations of the United States to found a new city in New Mexico for the purpose of caring especially for such members of the various fraternal organizations as may be affected with pulmonary tuberculosis. The purchase contemplates upward of 50,000 acres of land at an altitude of from 4,300 to 9,000 feet, and with the full facilities for caring for 50,000 patients. This "Fraternal City," as it will be called, will be, says *American Medicine,* on the municipical ownership plan, and stock-raising and garden-trucking well be offered to those desiring to work while recovering their health. At the head of the project, as President and Chairman of the Executive Committee, is W. R. Eidson, President of the Associated Fraternities of America, representing 3,000,000 members. A tax of a cent per month per capita has been fixed as the amount to be placed against the various fraternalists in the country. The direct location of the city has not yet been determined, but it is reported that the influence of two rival railroads is being pushed to the limit to secure favorable consideration, and that one prominent railroad officer has pledged $100,000 in cash if the sanatorium is located anywhere along his line.

